# 4-Acetyl-3,3-diethyl-5-hydr­oxy-2-morpholino-2,3-dihydro-1-benzofuran

**DOI:** 10.1107/S1600536808034065

**Published:** 2008-11-13

**Authors:** Andrés Vega, Oney Ramírez-Rodríguez, Maximiliano Martínez-Cifuentes, Andrés Ibañez, Ramiro Araya-Maturana

**Affiliations:** a Universidad Andres Bello, Facultad de Ecología y Recursos Naturales, Departamento de Química, Chile; b Departamento de Química Orgánica, Facultad de Ciencias Químicas y Farmacéuticas, Universidad de Chile, Chile; c Centro de Investigación Interdisciplinaria, Avanzada en Ciencia de los Materiales, Universidad de Chile, Chile

## Abstract

In the title compound, C_18_H_25_NO_4_, the benzofuran ring is almost planar and the morpholino ring displays a chair conformation. The packing of compound has a one-dimensional structure constructed through inter­molecular O—H⋯O hydrogen bonds. The conformation is stabilized by intra­molecular C—H⋯N and C—H⋯O inter­actions.

## Related literature

For biological activity, see: Araya-Maturana *et al.* (2002 ▶, 2006 ▶). For related structures, see: Dusausoy *et al.* (1973 ▶); Filarowski *et al.* (2005 ▶); Huang *et al.* (2004 ▶). For the synthesis, see: Castro *et al.* (1983 ▶). For hydrogen bonding, see: Desiraju (2002 ▶). For puckering parameters, see: Cremer & Pople, 1975 ▶).
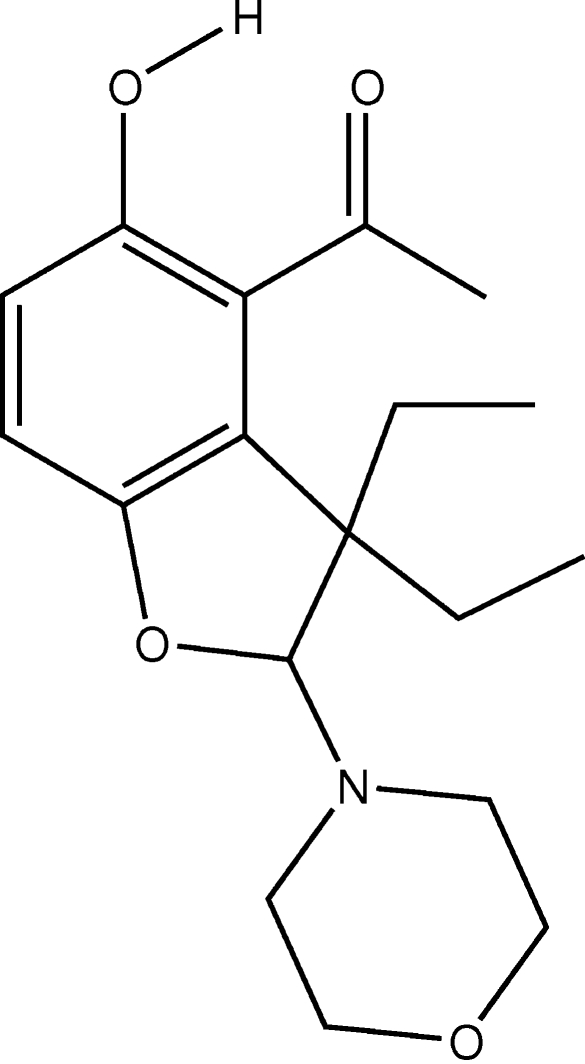


## Experimental

### 

#### Crystal data

C_18_H_25_NO_4_
*M* *_r_* = 319.39
Orthorhombic, 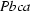

*a* = 7.7769 (2) Å
*b* = 19.4256 (5) Å
*c* = 22.3875 (6) Å
*V* = 3382.10 (15) Å^3^
*Z* = 8
Mo *K*α radiation
μ = 0.09 mm^−1^
*T* = 150 (2) K
0.43 × 0.30 × 0.30 mm

#### Data collection

Siemens SMART CCD area-detector diffractometer
Absorption correction: multi-scan (*SADABS*; Bruker 1999 ▶) *T*_min_ = 0.963, *T*_max_ = 0.974
19635 measured reflections
2988 independent reflections
2537 reflections with *I* > 2σ(*I*)
*R*_int_ = 0.027

#### Refinement

*R*[*F* ^2^ > 2σ(*F* ^2^)] = 0.037
*wR*(*F* ^2^) = 0.099
*S* = 1.04
2988 reflections
215 parameters
H atoms treated by a mixture of independent and constrained refinement
Δρ_max_ = 0.22 e Å^−3^
Δρ_min_ = −0.15 e Å^−3^

### 

Data collection: *SMART-NT* (Bruker, 2001 ▶); cell refinement: *SAINT-NT* (Bruker, 1999 ▶); data reduction: *SAINT-NT*; program(s) used to solve structure: *SHELXS97* (Sheldrick, 2008 ▶); program(s) used to refine structure: *SHELXL97* (Sheldrick, 2008 ▶); molecular graphics: *SHELXTL-NT* (Sheldrick, 2008 ▶); software used to prepare material for publication: *SHELXTL-NT*.

## Supplementary Material

Crystal structure: contains datablocks I, global. DOI: 10.1107/S1600536808034065/pv2104sup1.cif

Structure factors: contains datablocks I. DOI: 10.1107/S1600536808034065/pv2104Isup2.hkl

Additional supplementary materials:  crystallographic information; 3D view; checkCIF report

## Figures and Tables

**Table 1:** Hydrogen-bond geometry (Å, °)

*D*—H⋯*A*	*D*—H	H⋯*A*	*D*⋯*A*	*D*—H⋯*A*
O2—H2⋯O4^i^	0.90 (2)	1.86 (2)	2.7639 (14)	177 (2)
C11—H11*A*⋯N1	0.98	2.61	3.205 (2)	119
C12—H12*A*⋯O3	0.99	2.54	3.333 (2)	137
C15—H15*C*⋯O2	0.98	2.46	3.037 (2)	118

Symmetry code: (i) 
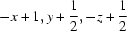
.

**Fig. 1.**
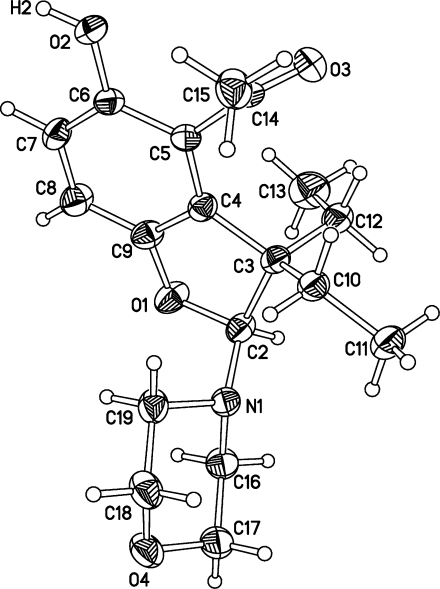
Molecular structure of (I) showing numbering scheme. Displacement ellipsoids have been plotted at 50% probability level and H atoms are shown as spheres of arbitrary radii.

**Fig. 2.**
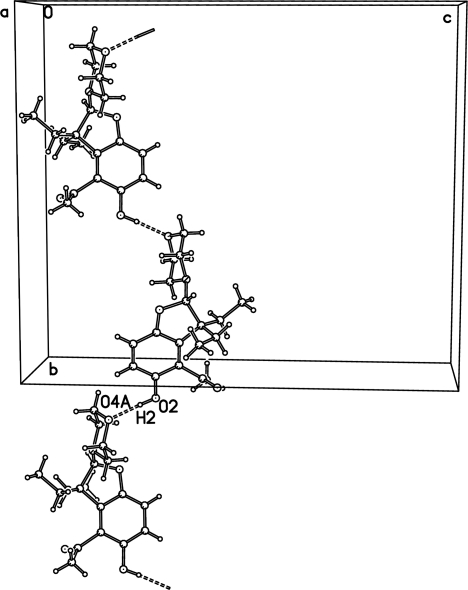
A view of the unit cell down the a-axis showing intermolecular hydrogen bonds leading to the formation of zigzag chains along the b-axis. Symmetry code: A = -x + 1, y + 1/2, -z + 1/2.

**Fig. 3.**
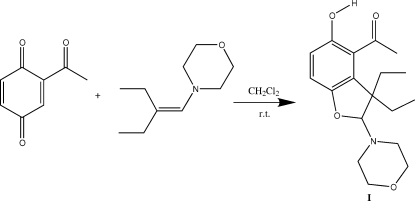
The formation of the title compound.

## supplementary crystallographic information

### Comment

The title compound, (I), is a structural model of its dimethyl analog (4-acetyl-3,3-dimethyl-5-hydroxy-2-morpholino-2,3-dihidrobenzo[*b*] furan). The later compound has been reported to be inactive as inhibitor of cellular respiration (Araya-Maturana *et al.*, 2002), despite the close analogy with inhibitors incorporating a carbonyl group in the *ortho* position with respect to a phenol function (Araya-Maturana *et al.*, 2002). The lack of activity has been atributed to the non-planarity of the acetyl group with respect to the phenolic moiety. Additionally, the O,*N*-acetal moiety of the molecule allows its use as starting point for the synthesis of biologically active quinones and hydroquinones (Araya-Maturana *et al.*, 2006).

The molecule of (I) displays a 2,3-dihydrobenzo[*b*]furanic skeleton, substituted at position 5 with an hydroxy group. A morpholino, an acetyl and two *gem* ethyl groups at positions 2, 4 and 3,3 respectively, are also present in the molecule (Fig. 1). While the aromatic ring is essentially planar, as expected by the π-conjugation, the furan ring is far from being planar, C2 is 0.331 (2) Å out of the plane formed by the remaining atoms in the ring, giving it an envelop conformation. The morpholino ring displays a classical chair conformation with puckering parameters (Cremer & Pople, 1975): *Q* = 0.580 (2) Å, θ = 1.88 (14)° and φ = 161 (5)°.

Surprisingly, the acetyl group at position 4 is not coplanar with the aromatic ring; the dihedral angle between the two least-squares planes is 66.84 (6)°, precluding the formation of an intramolecular hydrogen bond with the hydroxy group at position 5. This differs from the observation made in molecules like 2-hydroxy-6-methoxyacetophenone (Filarowski *et al.*, 2005) or 2,6-di-hydroxy-acetophenone (Huang *et al.*, 2004) where both, the acetyl and the phenyl ring are almost coplanar and, a rather strong (Desiraju, 2002) intramolecular hydrogen bond to the hydroxo group is defined. This is the case still for η^6^-2-hydroxyacetophenone-tricarbonylcrommium(0) (Dusausoy *et al.*, 1973) where coordination to the metal could withdraw electron density from the aromatic ring, weakening conjugation to the acetyl carbonyl group.

It is interesting to note that the non-planarity have been previously predicted from solution data (Araya-Maturana *et al.*, 2002), suggesting the steric repulsion with the gem ethyl groups at position 3 is stronger than the intramolecular hydrogen bond. Finally, the analysis establishes that the conformation of the molecule is preserved at this respect in solution.

The packing of the molecule displays an intermolecular hydrogen bond between hydroxy hydrogen atom and the morpholino oxygen atom of an adjacent molecule (*-x + 1, y + 1/2, -z + 1/2*), with O···O of 2.764 (1) Å. This head to tail interaction leads to the formation of zigzag chains along the *b*-axis (Fig. 2). The structure is stabilized by intramolecular interactions of the types C—H···N and C—H···O (details are given in Table 1).

The structure detemination supports the hypothesis that relates the lack of activity of the compound as a cell respiration inhibitor with the non planarity of the acetyl group in relation to the phenolic core.

### Experimental

The title compound was prepared from reaction of 4-(2-ethyl-but-1-enyl)-morpholine with 2-acetyl-1,4-benzoquinone (see Scheme 2), following the procedure described for the 3,3-dimethyl analog (Castro *et al.*, 1983). The reaction time was 2 h. X-ray quality crystals were obtained from the resulting solution after volume reduction and addition of a few drops of methanol.

### Refinement

The hydrogen atoms were included in the refinements at geometrically idealized positions using a riding model, with C—H distances in the range 0.96 to 1.00 Å and *U*_iso_(H) values were set equal to 1.5*U*_eq_ of the parent carbon atom for methyl groups and 1.2*U*_eq_ for the others. The hydroxyl hydrogen atom was located in a difference Fourier map and refined without any constrain.

### Crystal data

**Table:**

C_18_H_25_NO_4_	*F*_000_ = 1376
*M**_r_* = 319.39	*D*_x_ = 1.255 Mg m^−^^3^
Orthorhombic, *P**b**c**a*	Mo *K*α radiation λ = 0.71073 Å
Hall symbol: -P 2ac 2ab	Cell parameters from 6371 reflections
*a* = 7.7769 (2) Å	θ = 2.3–24.8º
*b* = 19.4256 (5) Å	µ = 0.09 mm^−^^1^
*c* = 22.3875 (6) Å	*T* = 150 (2) K
*V* = 3382.10 (15) Å^3^	Block, colorless
*Z* = 8	0.43 × 0.30 × 0.30 mm

### Data collection

**Table:**

Siemens SMART CCD area-detector diffractometer	2988 independent reflections
Radiation source: fine-focus sealed tube	2537 reflections with *I* > 2σ(*I*)
Monochromator: graphite	*R*_int_ = 0.027
*T* = 150(2) K	θ_max_ = 25.0º
φ and ω scans	θ_min_ = 1.8º
Absorption correction: part of the refinement model (Δ*F*)(SADABS; Bruker 1999)	*h* = −9→9
*T*_min_ = 0.963, *T*_max_ = 0.974	*k* = −23→23
19635 measured reflections	*l* = −26→26

### Refinement

**Table:**

Refinement on *F*^2^	Secondary atom site location: difference Fourier map
Least-squares matrix: full	Hydrogen site location: inferred from neighbouring sites
*R*[*F*^2^ > 2σ(*F*^2^)] = 0.037	H atoms treated by a mixture of independent and constrained refinement
*wR*(*F*^2^) = 0.099	*w* = 1/[σ^2^(*F*_o_^2^) + (0.0508*P*)^2^ + 1.1025*P*] where *P* = (*F*_o_^2^ + 2*F*_c_^2^)/3
*S* = 1.04	(Δ/σ)_max_ = 0.001
2988 reflections	Δρ_max_ = 0.22 e Å^−^^3^
215 parameters	Δρ_min_ = −0.15 e Å^−^^3^
Primary atom site location: structure-invariant direct methods	Extinction correction: none

### Special details

**Table:**

Experimental. Proton and ^13^C NMR spectra were acquired using a Bruker AVANCE DRX 300 spectrometer operating at 300.13 MHz (1*H*) or 75.47 MHz (13 C). All measurements were carried out at a probe temperature of 300 K.^1^H-RMN(CDCl_3_): 0.65(3*H*, t, *J* = 7 Hz); 1.05(3*H*, t, *J* = 7 Hz); 1.59–1.85 (4*H*, m, 2XCH_2_-CH_3_); 2.55(3*H*, S, CH_3_CO); 2.51–2.61(2*H*, m, CH_2_); 2.66–2.78(2*H*, m, CH_2_); 3.55–3.70(4*H*, m, 2XCH_2_); 4.71(1*H*, s, CH); 6.19(1*H*, s broad, OH); 6.56(1*H*, d, *J* = 8.5 Hz); 6.62(1*H*, d, _J_ = 8.5 Hz).^13^C-RMN(CDCl_3_): 8.36; 10.18; 25.45; 32.68; 32.90; 49.81; 52.00; 66.93; 106.51; 109.63; 115.36; 126.63; 129.92; 146.31; 153.25; 205.62.
Geometry. All e.s.d.'s (except the e.s.d. in the dihedral angle between two l.s. planes) are estimated using the full covariance matrix. The cell e.s.d.'s are taken into account individually in the estimation of e.s.d.'s in distances, angles and torsion angles; correlations between e.s.d.'s in cell parameters are only used when they are defined by crystal symmetry. An approximate (isotropic) treatment of cell e.s.d.'s is used for estimating e.s.d.'s involving l.s. planes.Least-squares planes (*x*,*y*,*z* in crystal coordinates) and deviations from them (* indicates atom used to define plane)7.0500 (0.0020) *x* + 7.3246 (0.0105) *y* + 4.2493 (0.0126) *z* = 12.9211 (0.0092)* -0.0040 (0.0010) C4 * 0.0120 (0.0009) C5 * -0.0103 (0.0010) C6 * 0.0006 (0.0010) C7 * 0.0076 (0.0011) C8 * -0.0059 (0.0010) C9Rms deviation of fitted atoms = 0.00781.4062(0.0062) *x* + 16.5869(0.0074) *y* - 10.9267(0.0135) *z* = 13.1527(0.0123)Angle to previous plane (with approximate e.s.d.) =S 66.84 (0.06)* 0.0007 (0.0003) C5 * -0.0022 (0.0011) C14 * 0.0007 (0.0003) C15 * 0.0009 (0.0004) O3
Refinement. Refinement of *F*^2^ against ALL reflections. The weighted *R*-factor *wR* and goodness of fit *S* are based on *F*^2^, conventional *R*-factors *R* are based on *F*, with *F* set to zero for negative *F*^2^. The threshold expression of *F*^2^ > σ(*F*^2^) is used only for calculating *R*-factors(gt) *etc*. and is not relevant to the choice of reflections for refinement. *R*-factors based on *F*^2^ are statistically about twice as large as those based on *F*, and *R*- factors based on ALL data will be even larger.

### Fractional atomic coordinates and isotropic or equivalent isotropic displacement parameters (Å^2^)

**Table:**

	*x*	*y*	*z*	*U*_iso_*/*U*_eq_	
O1	0.80326 (14)	0.80844 (5)	0.31337 (4)	0.0345 (3)	
C2	0.74318 (19)	0.78750 (7)	0.37275 (6)	0.0280 (3)	
H20	0.8428	0.7659	0.3939	0.034*	
N1	0.60986 (15)	0.73632 (6)	0.36854 (5)	0.0270 (3)	
C16	0.6798 (2)	0.66749 (7)	0.35651 (7)	0.0328 (3)	
H16A	0.7712	0.6567	0.3859	0.039*	
H16B	0.7314	0.6664	0.3161	0.039*	
C17	0.5395 (2)	0.61465 (8)	0.36064 (7)	0.0360 (4)	
H17A	0.5879	0.5684	0.3527	0.043*	
H17B	0.4913	0.6146	0.4016	0.043*	
O4	0.40535 (14)	0.62893 (5)	0.31851 (5)	0.0367 (3)	
C18	0.3362 (2)	0.69617 (7)	0.32890 (7)	0.0355 (4)	
H18A	0.2827	0.6976	0.3690	0.043*	
H18B	0.2457	0.7059	0.2990	0.043*	
C19	0.47396 (19)	0.75074 (8)	0.32496 (6)	0.0320 (3)	
H19A	0.5232	0.7514	0.2842	0.038*	
H19B	0.4232	0.7965	0.3331	0.038*	
C3	0.69878 (18)	0.85594 (7)	0.40661 (6)	0.0264 (3)	
C10	0.53656 (19)	0.85133 (7)	0.44592 (6)	0.0292 (3)	
H10A	0.5118	0.8978	0.4619	0.035*	
H10B	0.4385	0.8380	0.4202	0.035*	
C11	0.5450 (2)	0.80105 (8)	0.49828 (7)	0.0399 (4)	
H11A	0.5757	0.7552	0.4835	0.060*	
H11B	0.4327	0.7991	0.5180	0.060*	
H11C	0.6321	0.8167	0.5269	0.060*	
C12	0.85520 (19)	0.87683 (8)	0.44551 (7)	0.0328 (3)	
H12A	0.8271	0.9205	0.4661	0.039*	
H12B	0.8713	0.8412	0.4767	0.039*	
C13	1.0247 (2)	0.88622 (10)	0.41292 (8)	0.0492 (4)	
H13A	1.0583	0.8427	0.3942	0.074*	
H13B	1.1136	0.9004	0.4414	0.074*	
H13C	1.0117	0.9217	0.3821	0.074*	
C4	0.67804 (17)	0.90534 (7)	0.35435 (6)	0.0248 (3)	
C5	0.61288 (17)	0.97222 (7)	0.35094 (6)	0.0239 (3)	
C14	0.55496 (19)	1.01250 (7)	0.40490 (6)	0.0262 (3)	
O3	0.65792 (14)	1.02913 (5)	0.44311 (4)	0.0361 (3)	
C15	0.3689 (2)	1.03120 (8)	0.40908 (7)	0.0356 (4)	
H15A	0.3477	1.0554	0.4468	0.053*	
H15B	0.2990	0.9893	0.4077	0.053*	
H15C	0.3381	1.0612	0.3755	0.053*	
C6	0.60992 (18)	1.00459 (7)	0.29479 (6)	0.0257 (3)	
O2	0.54332 (14)	1.07011 (5)	0.29248 (5)	0.0315 (3)	
C7	0.67545 (19)	0.97197 (7)	0.24488 (6)	0.0303 (3)	
H7	0.6743	0.9951	0.2075	0.036*	
C8	0.7427 (2)	0.90606 (7)	0.24851 (7)	0.0333 (3)	
H8	0.7885	0.8837	0.2143	0.040*	
C9	0.74103 (18)	0.87401 (7)	0.30338 (6)	0.0284 (3)	
H2	0.562 (3)	1.0879 (10)	0.2559 (10)	0.063 (6)*	

### Atomic displacement parameters (Å^2^)

**Table:**

	*U*^11^	*U*^22^	*U*^33^	*U*^12^	*U*^13^	*U*^23^
O1	0.0429 (6)	0.0272 (5)	0.0333 (6)	0.0089 (5)	0.0125 (5)	0.0046 (4)
C2	0.0319 (7)	0.0258 (7)	0.0265 (7)	0.0034 (6)	0.0022 (6)	0.0045 (6)
N1	0.0327 (6)	0.0213 (6)	0.0270 (6)	0.0044 (5)	−0.0028 (5)	0.0003 (5)
C16	0.0385 (8)	0.0254 (7)	0.0344 (8)	0.0067 (6)	−0.0023 (7)	−0.0021 (6)
C17	0.0466 (9)	0.0255 (7)	0.0359 (8)	0.0036 (7)	−0.0054 (7)	−0.0019 (6)
O4	0.0447 (6)	0.0306 (6)	0.0349 (6)	0.0018 (5)	−0.0063 (5)	−0.0089 (4)
C18	0.0387 (8)	0.0343 (8)	0.0334 (8)	0.0064 (7)	−0.0059 (7)	−0.0076 (6)
C19	0.0398 (8)	0.0288 (7)	0.0274 (7)	0.0075 (7)	−0.0043 (6)	−0.0005 (6)
C3	0.0305 (7)	0.0222 (7)	0.0263 (7)	0.0007 (6)	0.0004 (6)	0.0026 (5)
C10	0.0362 (8)	0.0248 (7)	0.0265 (7)	−0.0007 (6)	0.0046 (6)	0.0005 (6)
C11	0.0569 (10)	0.0318 (8)	0.0311 (8)	−0.0051 (7)	0.0082 (7)	0.0046 (6)
C12	0.0366 (8)	0.0286 (8)	0.0333 (8)	−0.0005 (6)	−0.0056 (6)	0.0051 (6)
C13	0.0362 (9)	0.0511 (11)	0.0603 (11)	−0.0074 (8)	−0.0052 (8)	0.0048 (9)
C4	0.0241 (7)	0.0252 (7)	0.0252 (7)	−0.0020 (5)	0.0009 (5)	0.0029 (5)
C5	0.0245 (7)	0.0216 (7)	0.0257 (7)	−0.0044 (5)	0.0000 (5)	0.0007 (5)
C14	0.0360 (8)	0.0176 (6)	0.0249 (7)	−0.0042 (6)	−0.0015 (6)	0.0031 (5)
O3	0.0437 (6)	0.0339 (6)	0.0306 (6)	−0.0042 (5)	−0.0063 (5)	−0.0040 (4)
C15	0.0388 (9)	0.0380 (8)	0.0301 (8)	0.0050 (7)	0.0022 (6)	−0.0037 (6)
C6	0.0279 (7)	0.0207 (7)	0.0284 (7)	−0.0036 (6)	−0.0025 (6)	0.0022 (5)
O2	0.0446 (6)	0.0210 (5)	0.0288 (5)	0.0013 (4)	−0.0005 (5)	0.0042 (4)
C7	0.0374 (8)	0.0304 (7)	0.0231 (7)	−0.0019 (6)	0.0032 (6)	0.0066 (6)
C8	0.0406 (8)	0.0314 (8)	0.0278 (7)	0.0028 (6)	0.0106 (7)	0.0008 (6)
C9	0.0301 (7)	0.0238 (7)	0.0314 (7)	0.0023 (6)	0.0055 (6)	0.0018 (6)

### Geometric parameters (Å, °)

**Table:**

O1—C9	1.3808 (17)	C11—H11A	0.9800
O1—C2	1.4665 (16)	C11—H11B	0.9800
C2—N1	1.4395 (18)	C11—H11C	0.9800
C2—C3	1.5690 (19)	C12—C13	1.518 (2)
C2—H20	1.0000	C12—H12A	0.9900
N1—C19	1.4654 (18)	C12—H12B	0.9900
N1—C16	1.4683 (17)	C13—H13A	0.9800
C16—C17	1.501 (2)	C13—H13B	0.9800
C16—H16A	0.9900	C13—H13C	0.9800
C16—H16B	0.9900	C4—C9	1.3828 (19)
C17—O4	1.4338 (18)	C4—C5	1.3966 (19)
C17—H17A	0.9900	C5—C6	1.4057 (18)
C17—H17B	0.9900	C5—C14	1.5082 (18)
O4—C18	1.4315 (17)	C14—O3	1.2154 (17)
C18—C19	1.510 (2)	C14—C15	1.494 (2)
C18—H18A	0.9900	C15—H15A	0.9800
C18—H18B	0.9900	C15—H15B	0.9800
C19—H19A	0.9900	C15—H15C	0.9800
C19—H19B	0.9900	C6—O2	1.3752 (16)
C3—C4	1.5218 (18)	C6—C7	1.382 (2)
C3—C10	1.5409 (19)	O2—H2	0.90 (2)
C3—C12	1.550 (2)	C7—C8	1.385 (2)
C10—C11	1.5270 (19)	C7—H7	0.9500
C10—H10A	0.9900	C8—C9	1.377 (2)
C10—H10B	0.9900	C8—H8	0.9500
			
C9—O1—C2	106.92 (10)	C10—C11—H11A	109.5
N1—C2—O1	111.21 (11)	C10—C11—H11B	109.5
N1—C2—C3	117.28 (11)	H11A—C11—H11B	109.5
O1—C2—C3	105.85 (10)	C10—C11—H11C	109.5
N1—C2—H20	107.4	H11A—C11—H11C	109.5
O1—C2—H20	107.4	H11B—C11—H11C	109.5
C3—C2—H20	107.4	C13—C12—C3	116.30 (13)
C2—N1—C19	115.54 (11)	C13—C12—H12A	108.2
C2—N1—C16	111.96 (11)	C3—C12—H12A	108.2
C19—N1—C16	108.62 (11)	C13—C12—H12B	108.2
N1—C16—C17	110.01 (12)	C3—C12—H12B	108.2
N1—C16—H16A	109.7	H12A—C12—H12B	107.4
C17—C16—H16A	109.7	C12—C13—H13A	109.5
N1—C16—H16B	109.7	C12—C13—H13B	109.5
C17—C16—H16B	109.7	H13A—C13—H13B	109.5
H16A—C16—H16B	108.2	C12—C13—H13C	109.5
O4—C17—C16	110.86 (12)	H13A—C13—H13C	109.5
O4—C17—H17A	109.5	H13B—C13—H13C	109.5
C16—C17—H17A	109.5	C9—C4—C5	119.53 (12)
O4—C17—H17B	109.5	C9—C4—C3	108.62 (12)
C16—C17—H17B	109.5	C5—C4—C3	131.84 (12)
H17A—C17—H17B	108.1	C4—C5—C6	118.09 (12)
C18—O4—C17	110.06 (11)	C4—C5—C14	123.18 (12)
O4—C18—C19	111.39 (12)	C6—C5—C14	118.64 (12)
O4—C18—H18A	109.4	O3—C14—C15	121.94 (13)
C19—C18—H18A	109.4	O3—C14—C5	120.33 (13)
O4—C18—H18B	109.4	C15—C14—C5	117.73 (12)
C19—C18—H18B	109.4	C14—C15—H15A	109.5
H18A—C18—H18B	108.0	C14—C15—H15B	109.5
N1—C19—C18	109.80 (12)	H15A—C15—H15B	109.5
N1—C19—H19A	109.7	C14—C15—H15C	109.5
C18—C19—H19A	109.7	H15A—C15—H15C	109.5
N1—C19—H19B	109.7	H15B—C15—H15C	109.5
C18—C19—H19B	109.7	O2—C6—C7	122.20 (12)
H19A—C19—H19B	108.2	O2—C6—C5	116.99 (12)
C4—C3—C10	112.89 (11)	C7—C6—C5	120.79 (12)
C4—C3—C12	110.49 (11)	C6—O2—H2	109.1 (13)
C10—C3—C12	109.69 (11)	C6—C7—C8	121.04 (13)
C4—C3—C2	100.72 (10)	C6—C7—H7	119.5
C10—C3—C2	114.01 (11)	C8—C7—H7	119.5
C12—C3—C2	108.70 (11)	C9—C8—C7	117.80 (13)
C11—C10—C3	116.13 (13)	C9—C8—H8	121.1
C11—C10—H10A	108.3	C7—C8—H8	121.1
C3—C10—H10A	108.3	C8—C9—O1	123.93 (12)
C11—C10—H10B	108.3	C8—C9—C4	122.71 (13)
C3—C10—H10B	108.3	O1—C9—C4	113.36 (12)
H10A—C10—H10B	107.4		
			
O1—C2—C3—C4	19.89 (13)	C10—C3—C4—C9	−134.46 (13)
C2—C3—C4—C9	−12.49 (15)	C12—C3—C4—C9	102.28 (14)
C3—C4—C9—O1	0.16 (17)	C10—C3—C4—C5	46.6 (2)
N1—C16—C17—O4	59.38 (15)	C12—C3—C4—C5	−76.61 (19)
O4—C18—C19—N1	−58.28 (15)	C2—C3—C4—C5	168.61 (14)
C9—O1—C2—N1	107.54 (12)	C9—C4—C5—C6	1.7 (2)
C9—O1—C2—C3	−20.87 (14)	C3—C4—C5—C6	−179.54 (13)
O1—C2—N1—C19	−46.17 (15)	C9—C4—C5—C14	−174.88 (13)
C3—C2—N1—C19	75.82 (15)	C3—C4—C5—C14	3.9 (2)
O1—C2—N1—C16	78.87 (13)	C4—C5—C14—O3	64.89 (18)
C3—C2—N1—C16	−159.14 (12)	C6—C5—C14—O3	−111.63 (15)
C2—N1—C16—C17	172.46 (11)	C4—C5—C14—C15	−115.53 (15)
C19—N1—C16—C17	−58.76 (15)	C6—C5—C14—C15	67.95 (17)
C16—C17—O4—C18	−58.14 (16)	C4—C5—C6—O2	179.26 (12)
C17—O4—C18—C19	57.71 (15)	C14—C5—C6—O2	−4.04 (19)
C2—N1—C19—C18	−175.35 (11)	C4—C5—C6—C7	−2.3 (2)
C16—N1—C19—C18	57.90 (15)	C14—C5—C6—C7	174.40 (13)
N1—C2—C3—C4	−104.83 (13)	O2—C6—C7—C8	179.62 (13)
N1—C2—C3—C10	16.35 (17)	C5—C6—C7—C8	1.3 (2)
O1—C2—C3—C10	141.07 (11)	C6—C7—C8—C9	0.4 (2)
N1—C2—C3—C12	139.05 (12)	C7—C8—C9—O1	179.80 (13)
O1—C2—C3—C12	−96.23 (13)	C7—C8—C9—C4	−1.1 (2)
C4—C3—C10—C11	178.14 (12)	C2—O1—C9—C8	−167.31 (14)
C12—C3—C10—C11	−58.16 (16)	C2—O1—C9—C4	13.50 (16)
C2—C3—C10—C11	63.99 (16)	C5—C4—C9—C8	0.0 (2)
C4—C3—C12—C13	−52.74 (17)	C3—C4—C9—C8	−179.04 (14)
C10—C3—C12—C13	−177.83 (13)	C5—C4—C9—O1	179.21 (12)
C2—C3—C12—C13	56.91 (16)		

### Hydrogen-bond geometry (Å, °)

**Table:**

*D*—H···*A*	*D*—H	H···*A*	*D*···*A*	*D*—H···*A*
O2—H2···O4^i^	0.90 (2)	1.86 (2)	2.7639 (14)	177 (2)
C11—H11A···N1	0.98	2.61	3.205 (2)	119
C12—H12A···O3	0.99	2.54	3.333 (2)	137
C15—H15C···O2	0.98	2.46	3.037 (2)	118

Symmetry codes: (i) −*x*+1, *y*+1/2, −*z*+1/2.
